# Imaging and Clinical Parameters for Distinction between Infected and Non-Infected Fluid Collections in CT: Prospective Study Using Extended Microbiological Approach

**DOI:** 10.3390/diagnostics12020493

**Published:** 2022-02-14

**Authors:** Christopher Skusa, Romy Skusa, Moritz Wohlfarth, Philipp Warnke, Andreas Podbielski, Kristina Bath, Justus Groß, Clemens Schafmayer, Hagen Frickmann, Marc-André Weber, Andreas Hahn, Felix G. Meinel

**Affiliations:** 1 Institute of Diagnostic and Interventional Radiology, Pediatric Radiology and Neuroradiology, Rostock University Medical Center, 18057 Rostock, Germany; moritz.wohlfarth@uni-rostock.de (M.W.); kristina.bath@med.uni-rostock.de (K.B.); marc-andre.weber@med.uni-rostock.de (M.-A.W.); felix.meinel@med.uni-rostock.de (F.G.M.); 2 Institute of Medical Microbiology, Virology and Hygiene, Rostock University Medical Center, 18057 Rostock, Germany; romy.skusa@med.uni-rostock.de (R.S.); philipp.warnke@med.uni-rostock.de (P.W.); andreas.podbielski@med.uni-rostock.de (A.P.); frickmann@bnitm.de (H.F.); hahn.andreas@me.com (A.H.); 3 Department of General, Visceral, Vascular, Thoracic and Transplantation Surgery, Rostock University Medical Center, 18057 Rostock, Germany; justus.gross@med.uni-rostock.de (J.G.); clemens.schafmayer@med.uni-rostock.de (C.S.); 4 Department of Microbiology and Hospital Hygiene, Bundeswehr Hospital Hamburg, 20359 Hamburg, Germany

**Keywords:** CT, drainage, attenuation, gas entrapment, wall enhancement, abscess, infection

## Abstract

The aim of this investigation was to evaluate predictive CT imaging features and clinical parameters to distinguish infected from sterile fluid collections. Detection of infectious agents by advanced microbiological analysis was used as the reference standard. From April 2018 to October 2019, all patients undergoing CT-guided drainages were prospectively enrolled, if drainage material volume was at least 5 mL. Univariate analysis revealed attenuation (*p* = 0.001), entrapped gas (*p* < 0.001), fat stranding (*p* < 0.001), wall thickness (*p* < 0.001) and enhancement (*p* < 0.001) as imaging biomarkers and procalcitonin (*p* = 0.003) as clinical predictive parameters for infected fluid collections. On multivariate analysis, attenuation > 10 HU (*p* = 0.038), presence of entrapped gas (*p* = 0.027) and wall enhancement (*p* = 0.028) were independent parameters for distinguishing between infected and non-infected fluids. Gas entrapment had high specificity (93%) but low sensitivity (48%), while wall enhancement had high sensitivity (91%) but low specificity (50%). CT attenuation > 10 HU showed intermediate sensitivity (74%) and specificity (70%). Evaluation of the published proposed scoring systems did not improve diagnostic accuracy over independent predictors in our study. In conclusion, this prospective study confirmed that CT attenuation > 10 HU, entrapped gas and wall enhancement are the key imaging features to distinguish infected from sterile fluid collections on CT.

## 1. Introduction

Percutaneous drainage of infected fluid collections is recommended and generally accepted as the preferred treatment in eligible patients [1]. Percutaneous abscess drainage has a high therapeutic success rate and should be the therapy of choice because it is less invasive and less expensive than surgical drainage [2,3,4]. Technical improvements in computed tomography (CT) and ultrasound resulted in the increased use of this reliable and minimally invasive procedure. In addition to the therapeutic importance of CT, its role in diagnosing abscesses and other processes of infection is promising.

CT imaging parameters with predictive values for the presence of infected fluid accumulations include gas entrapment, fluid attenuation, fat stranding, wall enhancement, wall thickness and internal septa [5,6,7,8,9]. The sensitivity and specificity of these imaging findings are limited, making it difficult to distinguish sterile from non-sterile fluid collections. In CT-guided drainage of postoperative abdominal fluid collections, the detection rate for microorganisms is 48–78% [7,8,9]. Studies on image-guided drainage in suspected spondylodiscitis describe a lower detection rate of 27–65% [10,11,12,13]. Magnetic resonance imaging (MRI) is also gaining importance in the diagnosis of infectious foci. Thereby, better detection of pancreatic fluid [14], hepatic abscess [15], early spondylodiscitis [16] and brain abscess [17] can be shown for diffusion-weighted MRI.

Microbiological analysis is mandatory for diagnosis of deep-seated infections. The detection of microorganisms within the invasively obtained body fluids can either be associated with infection, i.e., with etiopathological relevance, or with colonization, and therefore questionable etiological relevance. In this study, we jointly investigated proof of replicable microbial pathogens either colonizing or infecting the invasively assessed body compartment. There is currently no gold standard for the pre-analytic parameters that are needed to obtain CT-guided drainage material. The culture approach in the case of clinical suspicion of intra-abdominal infection includes seeding on aerobic and anaerobic solid and liquid media [18]. For ascites, it has been shown that the detection rate can be increased by additional inoculation and incubation in blood cultures [19,20,21]. The same applies to the detection of bacteria in primary sterile materials [22,23,24]. The importance of molecular biological tests in microbiological diagnostics is also increasing due to their high sensitivity in the detection of microorganisms [25,26,27]. In order to estimate the true predictive value of imaging parameters, it seems essential to optimize the detection rate of microorganisms by means of suitable microbiological diagnostic approaches. Therefore, it seems reasonable to assume that the combination of several prediction criteria can increase the accuracy of diagnoses compared to a single criterion for differentiating infected from sterile collections.

Two scoring systems for postoperative abdominal fluid collections have been published. Gnannt et al. [6] proposed a system, hereinafter referred to as the “Gnannt score”, which includes the imaging parameters gas entrapment and CT attenuation and the clinical parameters diabetes and C-reactive protein (CRP) value, in order to differentiate between infected and non-infected postoperative fluid accumulation. Radosa et al. [7] also proposed a system, hereinafter referred to as the “Radosa score”, that improved the accuracy of differentiating fluid accumulations regarding the three CT imaging parameters (CT attenuation, gas entrapment and wall enhancement) and the clinical parameter C-reactive protein (CRP) value. For the development of a score, the gold standard used in these studies was microscopic or cultural detection of microorganisms [6,7]. From a radiological point of view, a scoring system that is common and easy to use is preferable.

The aim of our study was to evaluate CT images and clinical parameters in terms of their ability to differentiate between microorganisms containing abdominal and extra-abdominal fluids by an advanced microbiological approach. In addition, both existing scoring systems were applied to our study group with regard to their value for differentiating infected from non-infected fluid collections on the basis of clinical and imaging findings.

## 2. Materials and Methods

### 2.1. Study Design, Informed Consent and Institutional Review Board Approval

Approval was obtained from the responsible institutional review board (Ethics Committee, Faculty of Medicine, University of Rostock, Registration number A 2018-0138). Written informed consent was obtained from all participants prior to enrollment.

This prospective study was conducted between April 2018 and October 2019, and included 100 consecutive CT-guided drainages from foci suspicious for infection. Drainages were performed at the DIN EN ISO 9001-certified Institute of Diagnostic and Interventional Radiology, Pediatric Radiology and Neuroradiology, Rostock University Medical Center, Rostock, Germany. Patients were eligible for this study if they were referred for a clinically indicated CT-guided aspiration or drainage of one or more suspected infection foci. Exclusion criteria were an aspiration volume less than 5 mL (since this would not allow for reliable microbiological analysis using an extended approach) and patients under 18 years of age.

### 2.2. CT Imaging Prior to Drainage

**Figure 1:**
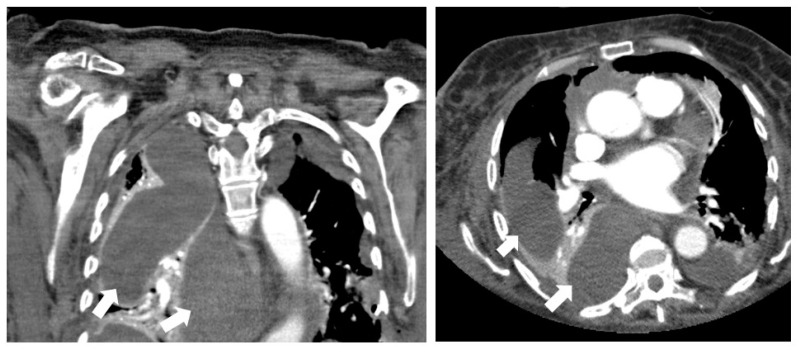
An 83-year-old woman with chambered pleural effusion (→) and CT attenuation of 8 HU. After percutaneous drainage, no pathogen was detected by microbiological approach.

**Figure 2:**
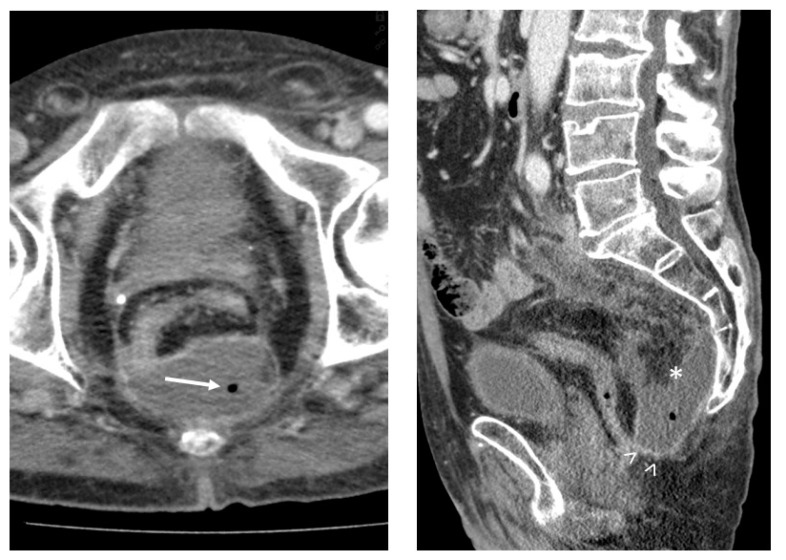
A 63-year-old man with presacral abscess (*), demonstrating gas entrapment (→), wall enhancement (>), fat stranding and CT attenuation of 29 HU. Microbiology after percutaneous drainage confirmed infection.

All CT studies were performed on a 64-detector CT scanner (Aquilion 64, Canon, Neuss, Germany). The contrast medium (iomeprol, Imeron 400 mg/mL, Bracco Imaging, Konstanz, Germany) was injected at a flow rate of 2.5 mL/s. CT data acquisition began in the portal venous phase of enhancement. Image reconstruction was performed using a medium-smooth soft tissue convolution kernel at a slice thickness of 0.5 mm. CT images were assessed using a picture archiving and communication system (PACS; IMPAX EE; Agfa, Moertel, Belgium). Based on previous scoring systems [6,7], the following imaging parameters were determined (Figure 1 and Figure 2):

- CT attenuation of the suspected fluid collection (in Hounsfield units (HU));
- Gas entrapment: the presence of superficial bubbles or air–fluid levels;
- Wall enhancement: contrast enhancement of the wall due to hypervascularization;
- Wall thickness: in case of encapsuled fluid collection, wall thickness was measured in millimeters;
- Fat stranding: increase in the attenuation of the surrounding adipose tissue.

CT data were analyzed by a radiologist with 5 years of experience in diagnostic radiology. Imaging parameters were recorded prospectively on the day of CT-guided drainage before microbiological results were available.

### 2.3. CT-Guided Drainage

CT-guided drainage was performed using standard techniques [28,29] covered by internal standard operating procedures. The ideal skin entry site was determined using planning scans with a radiopaque grid placed on the patient’s skin. The skin was then disinfected for 60 s according to the hospital hygiene standards using Braunoderm (Braun, Melsungen, Germany). After local anesthesia, a small nick in the skin was made and a 5 F Unidwell needle (Bard, Heidelberg, Germany) was advanced for fluid collection under CT guidance. Depending on the size of the fluid collection and the nature of the fluid, pigtail catheters with diameters ranging from 10 F to 16 F were chosen. The catheter was placed after the insertion of a wire and dilatation of the puncture tract to 1–2 F wider than the diameter of the pigtail catheter (OptiMed, Ettlingen, Germany). The content of the fluid was aspirated using a sterile syringe (BD Discardit II, Becton Dickinson GmbH, Heidelberg, Germany), then the drainage catheter was sutured to the skin and connected to a drainage bag (PFM medical, Köln, Germany).

### 2.4. Sample Transport and Processing

Blood culture bottles were inoculated on site by the radiologist. Therefore, BACTEC^TM^ Plus Aerobic/F and BACTEC^TM^ Lytic/10 Anaerobic/F blood culture media (Becton Dickinson GmbH, Heidelberg, Germany) were inoculated with 1 mL of the aspirated fluid each. The remaining sample material was left in the sterile syringe barrel. Blood culture bottles and the remaining sample material in the sterile syringe were then transported within 2 h at room temperature to the DIN EN ISO 15189-accredited Institute of Medical Microbiology, Virology and Hygiene of the University Medicine Rostock, Germany, for subsequent microbiological analyses.

### 2.5. Microbiological Analyses

To optimize the detection rate of microorganisms, we used an extended microbiological approach. The blood culture bottles were first added with 2 mL of BD BACTEC^TM^ FOS^TM^ Culture Supplement, then stored and incubated for up to five days in the BACTEC FX system (Becton Dickinson GmbH, Heidelberg, Germany), according the manufacturer’s recommendations. Naïve sample material underwent conventional routine culture-based growth of microorganisms that comprised incubation on aerobic and anaerobic agar and liquid media. Negative results in the conventional and blood culture methods were controlled by 16S ribosomal RNA (rRNA)- and 18S rRNA-gene PCR on bacterial and fungal DNA, respectively, performed from the original sample material. This microbiological approach was previously described in detail [30].

### 2.6. Laboratory and Clinical Parameters

Clinical data were retrieved from electronic medical records. Descriptive patients’ characteristics comprised age, sex, focus localization, underlying disease and referring unit. Laboratory results collected within 72 h of CT-guided intervention included C-reactive protein (CRP) in milligrams per liter, leukocytes in 10^3^ per microliter and procalcitonin in nanograms per milliliter. Clinical data were extracted from patient records: diagnosis of diabetes, current intake of immunosuppressive drugs or antibiotics, previous chemotherapy (within 6 months), previous operation and the presence of purulent specimen appearance.

### 2.7. Statistical Assessment

Data collection and statistical analyses were performed using SPSS software version 23 (Armonk, NY: IBM Corp.). Univariate analysis was performed using the χ^2^-test for categorical variables. The test of normal distribution for continuous variables was performed using the Shapiro–Wilk test, followed by univariate analysis using the t-test or the Mann–Whitney U test. The Youden index was used to identify optimal cutoff points for significant (*p*-value < 0.05) metric parameters of univariate analysis. After dichotomizing, binary logistic regression of all significant variables was performed, including a Hosmer–Lemeshow test. Finally, the data were evaluated by published scoring systems. The relevant parameters were compared using receiver operating characteristics (ROC) analysis with calculation of the area under the curve (AUC).

## 3. Results

### 3.1. Patient Characteristics

A total of 100 CT-guided drainages of 87 patients were included. The mean age was 63.1 years (SD ± 15.78). There were 59 biologically male and 28 biologically female patients. The most frequent patient characteristics were abdominal puncture localization (69%) with underlying primary diagnosis of infection or inflammation (41%), referred by general and visceral surgery (43%). All descriptive parameters of the study group are summarized in Table 1.

### 3.2. Univariate Comparison between Infected and Sterile Fluid Collections

In univariate analysis of imaging parameters, attenuation (*p* = 0.001), entrapped gas (*p* < 0.001), wall thickness (*p* < 0.001), wall enhancement (*p* < 0.001) and fat stranding (*p* < 0.001) differed significantly between the infected and sterile fluid collections. Of the clinical and laboratory parameters, only procalcitonin showed significant differences regarding infectious fluid specimens (Table 2).

For all numerical variables that showed significant differences between the microorganism-containing and sterile collections (*p* < 0.05), we calculated the optimal cutoff points using the Youden index. This dichotomization was performed in order to calculate and compare test characteristics. The optimal cutoff value for CT attenuation was 11 HU; for wall thickness, 1 mm; and for procalcitonin, 0.7 ng/mL. Test characteristics for all parameters with significant differences on univariate analysis are shown in Table 3. Among imaging parameters, gas entrapment within the fluid collection demonstrated the highest specificity (93%) but low sensitivity (48%), while wall enhancement showed high sensitivity (91%) but low specificity (50%). CT attenuation > 10 HU showed intermediate sensitivity (74%) and specificity (70%).

### 3.3. Multivariate Logistic Regression Analysis

**Figure 3:**
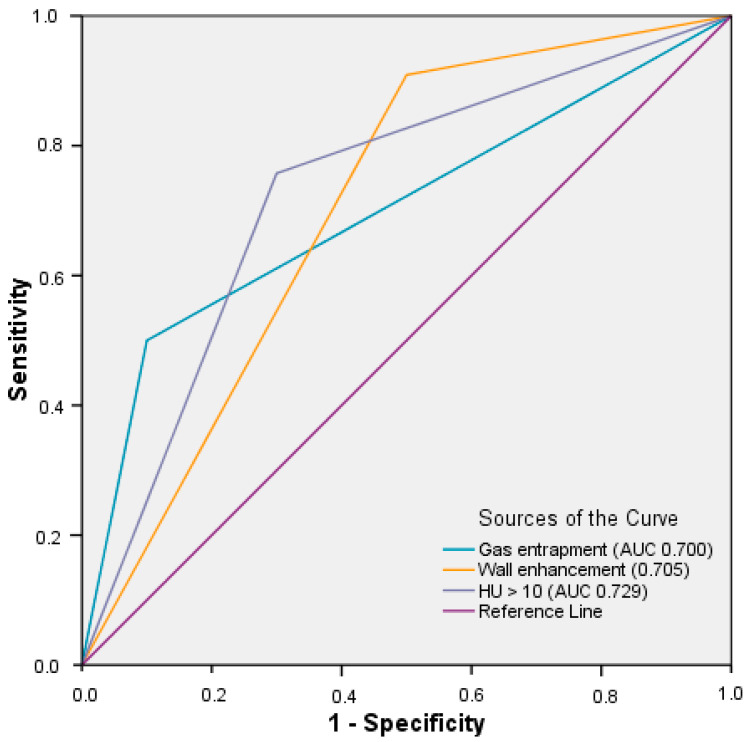
ROC curve for statistically significant parameters in multivariate regression analysis to identify microorganism-containing fluid collections applied to our cohort (n = 86).

Multivariate logistic regression analysis was performed to identify independent predictors of infected fluid collection. Because of the minimal measurable wall thickness as the cutoff point and the small number of patients with documented procalcitonin values (n = 25), these parameters were not included in multivariate binary logistic regression. The results of regression analysis are shown in Table 4. Multivariate regression analysis confirmed that gas entrapment, wall enhancement and CT attenuation were independent predictors for infected fluid collections. The Hosmer–Lemeshow test confirmed the goodness of fit for this logistic regression model (*p* = 0.990). ROC analysis of predictive parameters is shown in Figure 3.

### 3.4. Performance of Published Scoring Systems in Our Patient Cohort

**Figure 4:**
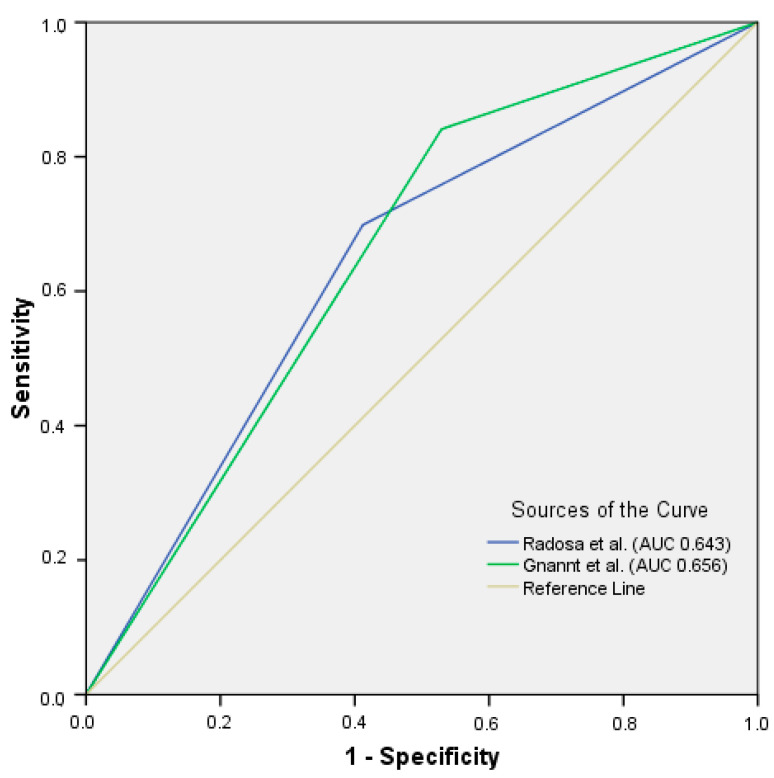
ROC curve for published scoring systems to identify microorganism-containing fluid collections, applied to our cohort.

We further applied two published scoring systems for distinguishing infected from sterile fluid collections by Gnannt et al. [6] and Radosa et al. [7] in order to validate and compare their diagnostic values in our cohort. The results of sensitivity, specificity, positive predictive value (PPV) and negative predictive value (NPV) are summarized in Table 5. Comparing test error results, 12/25 (48%) and 19/29 (65.5%) would have been assessed as false negative and 11/69 (15.9%) and 7/51 (13.7%) as false positive using the Gannt and Radosa score, respectively. A score value of ≥3 (Gnannt et al.) and ≥5 (Radosa et al.) as the cutoff was used for this calculation for the purpose of comparison. Both scores showed an AUC of 0.656 (95% CI 0.498–0.814) (Gnannt score) and 0.643 (95% CI 0.491–0.795) (Radosa score) (Figure 4).

## 4. Discussion

This prospective study demonstrated that the imaging parameters gas entrapment, wall enhancement and CT attenuation are independent predictors of infected fluid collections. The evaluation of recently published scoring systems by Gnannt et al. [6] and Radosa et al. [7] did not improve the differentiation between sterile and infected fluid collections beyond these individual parameters.

Previous studies by Gnannt et al. [6] and Radosa et al. [7] defined fluid collections as infected when Gram stain or culture revealed a positive result. This definition bears the method-inherent risk of false positive results due to artifact-related Gram stains and of false negative results by missing non-culturable microorganisms or by antibiotic pre-treatment. Therefore, the detection of an infectious agent by a microbiological diagnostic approach including cultural and molecular analyses was used as the reference standard in our study.

In accordance with both scoring systems and recent literature reports, the imaging parameters gas entrapment and CT attenuation were determined as predictors for the presence of infectious fluid collections based on our patient population [6,7,8]. When considering the proposed CT attenuation cutoff values of 10 to <20 HU (one score point) and >20 HU (two score points) by Gnannt et al., >20 HU by Radosa et al. and >10 HU from our patient population, it is noticeable that these values are in the same range. Due to the infection, cell debris accumulation leads to an increase in density correlated with greater X-ray absorption, consequently resulting in higher CT attenuation. Other studies confirm the value of CT attenuation in abscess diagnosis as a predictor for pyonephrosis [31,32]. The additional imaging parameter wall enhancement was not included in the Gnannt score, but was taken into account in the Radosa score. For the former, the presence of wall enhancement was able to successfully differentiate sterile from infectious fluid collections in the univariate analysis, but it was not included in the scoring system [6]. According to a study by Allen et al., there was no association between wall enhancement and the presence of infectious fluid [8].

While we found no clinical parameter to be a predictive factor of infected abdominal fluid collection, diabetes is considered a predictive factor in the Gnannt score [6]. Radosa et al. noticed the lack of information regarding medical history [7], which is supported by our data in which the status of pre-existing diabetes was recorded in a minority of patients. Studies have shown a correlation between diabetes and postoperative abdominal abscesses [33,34], but its predictive value in CT-guided diagnosis remains to be investigated.

In contrast to both scores by Gnannt et al. [6] and Radosa et al. [7], the CRP values did not significantly differ between sterile and infectious fluid collections in our patient population. Yet, in the univariate analysis (*p* = 0.096), there was a trend toward elevated CRP values when microorganism-containing fluid collections were detectable. The determination of CRP values is well-established in routine clinical practice as a marker of bacterial infections [35]. Studies have shown the predictive value of postoperative CRP measurement for complications in general, and specifically for the severity of complications [36,37,38]. Our univariate analysis suggested that procalcitonin provides better differentiation of microorganism-containing fluids (*p* = 0.003), but it could not be included in multivariate analysis because procalcitonin levels had only been measured in a minority of patients (n = 25). Previous studies have also shown an advantage of procalcitonin in comparison to CRP in bacterial infection diagnosis [39,40,41], and they may provide important perspectives in scoring systems.

While the two published scoring systems were established only for postoperative intra-abdominal abscesses, we extended our inclusion criteria to all suspicious infected fluid collections regardless of localization (thoracic, abdominal and musculoskeletal) and clinical setting (also including non-operative cases). Therefore, localization-independent imaging parameters could be found, simplifying CT-guided diagnosis of infected fluid collections. In our independent prospective study cohort, the published scoring systems did not improve diagnostic accuracy over individual CT imaging features alone. Thus, the use of simple imaging criteria appears to be sufficient and more suitable for routine assessment.

Nevertheless, our prospective study confirms that the diagnostic accuracy of imaging and laboratory parameters in distinguishing infected from sterile fluid collections remains imperfect. In particular, the absence of such imaging features does not reliably rule out infection. Therefore, the decision of whether to drain a fluid collection should not be based on imaging and/or laboratory parameters alone, but on the overall judgment of the clinical team, ideally in a multidisciplinary discussion.

As potential limitations of this study, the simultaneous inoculation of blood culture bottles might have led to a higher contamination rate. To minimize this risk, a rigorous hygienic regimen during CT-guided puncture was performed until we completed sampling. Due to our prospective study design, there was a limited number of included patients during the study period in comparison to previous retrospective studies. We focused our analysis on established imaging parameters that have been proposed in the literature as being helpful for differentiating infected from non-infected collections. Future studies are warranted to evaluate other imaging parameters such as the size, shape and texture of fluid collections.

## 5. Conclusions

In conclusion, the imaging parameters gas entrapment, wall enhancement and CT attenuation demonstrated their predictive value for the presence of infected fluids in our study population, including patients with different localization of foci in need of drainage and using an extended microbiological approach. The diagnostic value of labor parameters, especially procalcitonin, remains to be investigated in more detail. Published scoring systems did not show higher diagnostic accuracy in comparison with single predictive parameters.

## Figures and Tables

**Table 1:** Demographic data of patients with fluid collection.

Characteristics	
No. of CT-guided drainages performed (n_1_)	100
No. of patients (n_2_)	87
Age, years (±SD)	63 (±16)
Sex, male/female (n_2_ = 78)	59/28
Localization of fluid collection (n_1_ = 100)	
Thorax	21
Abdomen	69
Musculoskeletal	10
Underlying primary disease (n_1_ = 100)	
Carcinoma	38
Infection or inflammation	41
Vascular	13
Other	8
Referring unit (n_1_ = 100)	
General and visceral surgery	43
Intensive care unit	23
Internal medicine	20
Urology	5
Other surgical departments	8
Other departments	1

**Table 2:** CT imaging findings, laboratory results, and clinical data of patients with fluid collection.

Imaging Parameter	All Lesions (n = 100)	Infected Fluid Collection (n = 73)	Sterile Fluid Collection (n = 27)	*p*-Value
Attenuation, HU	Median (IQR)	14 (13)	5 (14)	**0.001**
Entrapped gas	Existent	35	2	**<0.001**
	None	38	25	
Wall thickness, mm	Median (IQR)	2.6 (1.55)	0 (2.2)	**<0.001**
Wall enhancement	Existent	60	10	**<0.001**
	Strong	30	5	
	Slight	30	5	
	None	6	10	
	Scan without contrast	7	7	
Fat stranding (without thorax, n = 79)	Existent	61	7	**<0.001**
	None	7	4	
Clinical and laboratory parameters				
CRP, mg/L (n = 94)	Median (IQR)	161 (145)	107 (187)	0.096
Leukocytes, 10^3^/µL (n = 99)	Median (IQR)	12.5 (7.7)	11.7 (7.9)	0.356
Procalcitonin, ng/mL (n = 25)	Median (IQR)	3.0 (28.0)	0.4 (0.3)	**0.003**
Diabetes (n = 27)		20	7	1
Immunosuppressive drugs (n = 15)		13	2	0.23
Chemotherapeutics (n = 14)		10	4	1
Previous antibiotic therapy (n = 95)				0.801
yes		50	18	
no		19	8	
Previous operation (n = 95)		49	18	1

HU—Hounsfield units; CRP—C-reactive protein.

**Table 3:** Test characteristics for parameters to identify microorganism-containing fluid collections.

Parameter	Sens	Spec	NPV	PPV	AUC
Fat stranding present (n = 100)	0.90	0.36	0.36	0.90	0.630
Gas entrapment present (n = 100)	0.48	0.93	0.40	0.95	0.703
Wall thickness > 1 mm (n = 100)	0.92	0.56	0.71	0.85	0.737
Wall enhancement present (n = 86)	0.91	0.50	0.63	0.86	0.705
CT attenuation > 10 HU (n = 100)	0.74	0.70	0.50	0.87	0.722

Sens—sensitivity; Spec—specificity; NPV—negative predictive value; PPV—positive predictive value; AUC—area under the curve.

**Table 4:** Multivariate analysis.

Parameter	β	*p*-Value	OR
Fat stranding Yes/no		0.906	
Gas entrapment Yes/no	1.830	0.027	6.234
Wall enhancement Yes/no	1.582	0.028	4.865
CT attenuation HU > 10 HU ≤ 10	1.343	0.038	3.832

β—regression coefficient; OR—odds ratio.

**Table 5:** Comparison of published scoring systems.

Applied Score	Sens	Spec	NPV	PPV	AUC
Gnannt score	0.84 [CI 0.77; 0.91]	0.47 [CI 0.37; 0.57]	0.44 [CI 0.34; 0.54]	0.86 [CI 0.78; 0.93]	0.656
Radosa score	0.70 [CI 0.60; 0.80]	0.59 [CI 0.48; 0.70]	0.35 [CI 0.24; 0.44]	0.86 [CI 0.79; 0.94]	0.643

Performance of published scoring systems in our patient cohort. Sens—sensitivity; Spec—specificity; NPV—negative predictive value; PPV—positive predictive value; AUC—area under the curve; CI—confidence interval.

## Data Availability

All supporting data are available from the corresponding author upon reasonable request.

## References

[1] Politano A.D., Hranjec T., Rosenberger L.H., Sawyer R.G., Tache Leon C.A. (2011). Differences in morbidity and mortality with percutaneous versus open surgical drainage of postoperative intra-abdominal infections: A review of 686 cases. Am. Surg..

[2] Van Sonnenberg E., Wittich G.R., Goodacre B.W., Casola G., D’Agostino H.B. (2001). Percutaneous abscess drainage: Update. World J. Surg..

[3] Benoist S., Panis Y., Pannegeon V., Soyer P., Watrin T., Boudiaf M., Valleur P. (2002). Can failure of percutaneous drainage of postoperative abdominal abscesses be predicted?. Am. J. Surg..

[4] Cinat M.E., Wilson S.E., Din A.M. (2002). Determinants for successful percutaneous image-guided drainage of intra-abdominal abscess. Arch. Surg..

[5] Çullu N., Kalemci S., Karakaş Ö., Eser İ., Yalçın F., Boyacı F.N., Karakaş E. (2013). Efficacy of CT in diagnosis of transudates and exudates in patients with pleural effusion. Diagn. Interv. Radiol..

[6] Gnannt R., Fischer M.A., Baechler T., Clavien P.-A., Karlo C., Seifert B., Lesurtel M., Alkadhi H. (2015). Distinguishing infected from noninfected abdominal fluid collections after surgery: An imaging, clinical, and laboratory-based scoring system. Investig. Radiol..

[7] Radosa C.G., Radosa J.C., Laniado M., Brandt J., Streitzig J., Seppelt D., Volk A., Plodeck V., Kühn J.P., Hoffmann R.-T. (2020). Infected versus sterile abdominal fluid collections in postoperative CT: A scoring system based on clinical and imaging findings. Abdom. Radiol..

[8] Allen B.C., Barnhart H., Bashir M., Nieman C., Breault S., Jaffe T.A. (2012). Diagnostic accuracy of intra-abdominal fluid collection characterization in the era of multidetector computed tomography. Am. Surg..

[9] Jaques P., Mauro M., Safrit H., Yankaskas B., Piggott B. (1986). CT features of intraabdominal abscesses: Prediction of successful percutaneous drainage. AJR Am. J. Roentgenol..

[10] Sertic M., Parkes L., Mattiassi S., Pritzker K., Gardam M., Murphy K. (2019). The Efficacy of Computed Tomography-Guided Percutaneous Spine Biopsies in Determining a Causative Organism in Cases of Suspected Infection: A Systematic Review. Can. Assoc. Radiol. J..

[11] Schwarz-Nemec U., Friedrich K.M., Stihsen C., Schwarz F.K., Trattnig S., Weber M., Grohs J.G., Nemec S.F. (2020). Vertebral Bone Marrow and Endplate Assessment on MR Imaging for the Differentiation of Modic Type 1 Endplate Changes and Infectious Spondylodiscitis. J. Clin. Med..

[12] Braun A., Germann T., Wünnemann F., Weber M.-A., Schiltenwolf M., Akbar M., Burkholder I., Kauczor H.-U., Rehnitz C. (2019). Impact of MRI, CT, and Clinical Characteristics on Microbial Pathogen Detection Using CT-Guided Biopsy for Suspected Spondylodiscitis. J. Clin. Med..

[13] Spira D., Germann T., Lehner B., Hemmer S., Akbar M., Jesser J., Weber M.-A., Rehnitz C. (2016). CT-Guided Biopsy in Suspected Spondylodiscitis—The Association of Paravertebral Inflammation with Microbial Pathogen Detection. PLoS ONE.

[14] Borens B., Arvanitakis M., Absil J., El Bouchaibi S., Matos C., Eisendrath P., Toussaint E., Deviere J., Bali M.A. (2017). Added value of diffusion-weighted magnetic resonance imaging for the detection of pancreatic fluid collection infection. Eur. Radiol..

[15] Schmid-Tannwald C., Schmid-Tannwald C.M., Morelli J.N., Neumann R., Reiser M.F., Nikolaou K., Rist C. (2014). Role of diffusion-weighted MRI in differentiation of hepatic abscesses from non-infected fluid collections. Clin. Radiol..

[16] Chen T.-Y., Wu T.-C., Tsui Y.-K., Chen H.-H., Lin C.-J., Lee H.-J., Wu T.-C. (2015). Diffusion-weighted magnetic resonance imaging and apparent diffusion coefficient mapping for diagnosing infectious spondylodiscitis: A preliminary study. J. Neuroimaging.

[17] Ady J., Fong Y. (2014). Imaging for Infection: From Visualization of Inflammation to Visualization of Microbes. Surg. Infect. (Larchmt).

[18] Expertengremium Mikrobiologisch-Infektiologische Qualitätsstandards (2012). Intraabdominelle Infektionen unter Besonderer Berücksichtigung der Peritonitis.

[19] Guarner C., Soriano G. (1997). Spontaneous bacterial peritonitis. Semin. Liver Dis..

[20] Sajjad M., Khan Z.A., Khan M.S. (2016). Ascitic Fluid Culture in Cirrhotic Patients with Spontaneous Bacterial Peritonitis. J. Coll. Physicians Surg. Pak..

[21] Rimola A., García-Tsao G., Navasa M., Piddock L.J., Planas R., Bernard B., Inadomi J.M. (2000). Diagnosis, treatment and prophylaxis of spontaneous bacterial peritonitis: A consensus document. J. Hepatol..

[22] Sorlin P., Mansoor I., Dagyaran C., Struelens M.J. (2000). Comparison of resin-containing BACTEC Plus Aerobic/F* medium with conventional methods for culture of normally sterile body fluids. J. Med. Microbiol..

[23] Cetin E.S., Kaya S., Demirci M., Aridogan B.C. (2007). Comparison of the BACTEC blood culture system versus conventional methods for culture of normally sterile body fluids. Adv. Ther..

[24] Akcam F.Z., Yayli G., Uskun E., Kaya O., Demir C. (2006). Evaluation of the Bactec microbial detection system for culturing miscellaneous sterile body fluids. Res. Microbiol..

[25] Gu W., Deng X., Lee M., Sucu Y.D., Arevalo S., Stryke D., Federman S., Gopez A., Reyes K., Zorn K. (2021). Rapid pathogen detection by metagenomic next-generation sequencing of infected body fluids. Nat. Med..

[26] Lampejo T., Ciesielczuk H., Lambourne J. (2021). Clinical utility of 16S rRNA PCR in pleural infection. J. Med. Microbiol..

[27] Bivand J.M., Nygaard R.M., Kommedal Ø. (2021). Characterization of abscesses from liver, pancreas and kidney using deep sequencing of the 16S rRNA gene. Diagn. Microbiol. Infect. Dis..

[28] Nöldge G., Richter G.M., Grenacher L., Brado M., Kauffmann G.W. (1996). CT-gesteuerte Punktionen. Radiologe.

[29] Commander C.W., Wilson S.B., Bilaj F., Isaacson A.J., Burke C.T., Yu H. (2020). CT-Guided Percutaneous Drainage Catheter Placement in the Abdomen and Pelvis: Predictors of Outcome and Protocol for Follow-up. J. Vasc. Interv. Radiol..

[30] Skusa R., Skusa C., Wohlfarth M., Hahn A., Frickmann H., Weber M.-A., Podbielski A., Warnke P. (2021). How to Handle CT-Guided Abscess Drainages in Microbiological Analyses? Sterile Vials vs. Blood Culture Bottles for Transport and Processing. Microorganisms.

[31] Yuruk E., Tuken M., Sulejman S., Colakerol A., Serefoglu E.C., Sarica K., Muslumanoglu A.Y. (2017). Computerized tomography attenuation values can be used to differentiate hydronephrosis from pyonephrosis. World J. Urol..

[32] Boeri L., Fulgheri I., Palmisano F., Lievore E., Lorusso V., Ripa F., D’Amico M., Spinelli M.G., Salonia A., Carrafiello G. (2020). Hounsfield unit attenuation value can differentiate pyonephrosis from hydronephrosis and predict septic complications in patients with obstructive uropathy. Sci. Rep..

[33] Ming P.C., Yan T.Y.Y., Tat L.H. (2009). Risk factors of postoperative infections in adults with complicated appendicitis. Surg. Laparosc. Endosc. Percutan. Tech..

[34] Trinh J.V., Chen L.F., Sexton D.J., Anderson D.J. (2009). Risk factors for gram-negative bacterial surgical site infection: Do allergies to antibiotics increase risk?. Infect. Control Hosp. Epidemiol..

[35] Healy B., Freedman A. (2006). Infections. BMJ.

[36] Abet E., Drissi F., Couëtte C., Jean M.-H., Denimal F., Podevin J., Duchalais E., Meurette G. (2020). Predictive value of inflammatory markers for postoperative recovery following colorectal surgery. Int. J. Colorectal Dis..

[37] McSorley S.T., Ramanathan M.L., Horgan P.G., McMillan D.C. (2015). Postoperative C-reactive protein measurement predicts the severity of complications following surgery for colorectal cancer. Int. J. Colorectal Dis..

[38] Warschkow R., Beutner U., Steffen T., Müller S.A., Schmied B.M., Güller U., Tarantino I. (2012). Safe and early discharge after colorectal surgery due to C-reactive protein: A diagnostic meta-analysis of 1832 patients. Ann. Surg..

[39] Carboni G.L., Fahrner R., Gazdhar A., Printzen G., Schmid R.A., Hoksch B. (2008). Comparison of procalcitonin and CrP in the postoperative course after lung decortication. Eur. J. Cardiothorac. Surg..

[40] Suberviola B., Rellan L., Riera J., Iranzo R., Garcia Campos A., Robles J.C., Vicente R., Miñambres E., Santibanez M. (2017). Role of biomarkers in early infectious complications after lung transplantation. PLoS ONE.

[41] Kapoor S. (2009). The rapidly expanding role of procalcitonin as a diagnostic and prognostic assay besides in UTIs. Int. Urol. Nephrol..

